# Epithelial Changes in the Testosterone-Dominant Vagina: Implications for Menopause, Transgender Care, and Beyond

**DOI:** 10.3390/cells15090745

**Published:** 2026-04-22

**Authors:** Sarah Montgomery, Robin R. Ingalls

**Affiliations:** 1 Section of Infectious Diseases, Department of Medicine, Boston Medical Center, Boston, MA 02118, USA; 2 School of Medicine, Boston University, Boston, MA 02218, USA

**Keywords:** vaginal immunity, testosterone, inflammation, hormone-driven transcriptomics

## Abstract

Hormonal fluctuations across female life stages drive numerous transcriptomic and epigenetic changes, yet the effects of sex hormones on mucosal immunity, particularly in the vaginal epithelium, remain poorly understood. The vaginal mucosa undergoes cyclical remodeling during the ovulatory cycle under the influence of estrogens and progesterone produced mainly in the ovary. The ovary can also be a source of testosterone, and in postmenopausal women, as well as transgender men receiving hormone therapy, phenotypic changes in the vagina due to increased testosterone have been observed. However, the consequences of testosterone dominance in this tissue in terms of resilience and inflammation have not been well characterized. The goal of this study was to identify the histological and immunological changes within the vaginal epithelial cell barrier in an estrogen- vs. testosterone-dominant environment using an established in vitro reconstructed vaginal epithelial tissue model. Compared to estradiol, exposure to testosterone resulted in a thinner tissue with alterations in the cornification, although no impairment in the epithelial barrier was detected. Each hormone also resulted in a unique RNA expression profile, including increased expression of tight junction genes and decreased expression of chemokines and their receptors in testosterone compared to estradiol exposure. These data have implications for women’s health, including menopause, transgender men using gender-affirming hormone therapy, and other conditions associated with high testosterone in the vaginal compartment.

## 1. Introduction

Biological sex-driven differences in immunity have long been known to exist (reviewed in [1]). For example, females exhibit a more robust antibody response to many vaccines compared to males [2], but they are more likely to develop autoimmune diseases [3] and suffer higher mortality rates from sepsis [4]. Females have also been found to have decreased rates of infection with some pathogens, including *Helicobacter pylori* [5], *Mycobacterium tuberculosis* [6], and hepatitis B [7]. More recently, data from the COVID-19 pandemic suggests that being female protects against death [8]. These observations indicate that biological sex is an important immunomodulator, although the exact basis of this remains unclear and may be multifactorial. For example, there are well-described transcriptomic and epigenetic changes associated with the hormonal shifts seen with puberty, pregnancy and menopause in females (reviewed in [1]). Data on the effects of sex hormones on immune cell function and cytokine induction is mixed, and it has been difficult to confirm in vivo observations with in vitro studies (reviewed in [9]). Even less is known about the immunologic effects of sex hormones on epithelial cells and mucosal surfaces.

The vaginal mucosa consists of stratified squamous epithelium renewed from the basal surface as cells migrate towards the lumen. The tissue can be divided into three distinct zones: the mitotically active basal layer (“stratum basale”) from which the tissue is generated; the superficial layer of flattened, cornified cells (stratum corneum), which lack nuclei and desquamate during the ovulatory cycle; and a middle layer of suprabasal epithelial cells that is the site of cornification for the stratum corneum [10,11]. The vaginal epithelium is normally non-keratinized. However, similar to skin, cornification at this site can occur, resulting in the flattening of cells due to terminal differentiation and cell death at the apical surface [12]. Epithelial cell proliferation and the thickness of the vaginal mucosa is regulated by cycling estrogen and progesterone throughout the ovulatory cycle [13]. Estrogen also promotes the production of intracellular glycogen, which is then released as cells slough off, where it can be metabolized by *Lactobacillus* spp., reducing the vaginal pH, and inhibiting the growth of anaerobic bacteria and STD pathogens [14,15]. In post-menopausal women, testosterone and androstenedione are synthesized at high concentrations in the ovary, with minimal estrogen secretion, leading to transitional cell metaplasia and senile atrophy of the vagina and exocervix [16,17]; this is believed to result in a thinner, more fragile vaginal mucosal surface. Similar findings have been observed in vaginal tissue from transgender men undergoing testosterone therapy as part of gender-affirming medical care, along with the development of prostatic-like glands and paratubal epididymis-like mesonephric hypertrophy [18]. Notably, there is growing interest in the use of testosterone replacement therapy in postmenopausal women with vaginal atrophy and/or hypoactive sexual desire, although the long-term effects have not been studied [19].

The importance of understanding the effects of hormonal changes on vaginal immune defenses cannot be underestimated. Women spend 40% of their lives in the post-menopausal phase. In the US, it is estimated that 2 million women reach menopause each year [20], and, globally, the estimate is 25 million [21]. Transgender individuals undergoing gender-affirming care would also benefit from an improved understanding of the effects of cross-sex hormones on the reproductive tract. A recent analysis by the Williams Institute estimates that more than 2 million people in the U.S., approximately 0.8% of the population, identify as transgender, divided almost evenly between transgender men, transgender women, and non-binary adults [22]. Older epidemiologic data from the U.S. and Canada put the estimates slightly lower, with 0.5–0.6% of adults identifying as transgender and the majority being transgender men [23,24,25].

Given the limited information on the effects of testosterone on vaginal mucosal health in both transgender men and postmenopausal women, we set out to examine the histological and transcriptional changes within the vaginal epithelium in an estrogen- vs. testosterone-dominant environment, using an established in vitro reconstructed tissue model. We found hormone-specific histological differences in the epithelium, as well as distinct gene expression profiles, using RNA sequencing. This demonstrates that sex hormone-directed changes occur in the vaginal epithelial cell microenvironment that impact steady-state innate immune defenses at this surface that could impact the tissue resilience and the response to sexually transmitted infections, including HIV.

## 2. Materials and Methods

### 2.1. 3D Vaginal Tissues and Cell Culture

EpiVaginal reconstructed tissues were purchased from the MatTek Corporation (Andover, MA, USA). Briefly, these tissues were derived from cryopreserved primary vaginal–ectocervical epithelial cells from healthy women donors of reproductive age, and seeded onto cell culture inserts and grown at the air–liquid interface (ALI). VEC-100 partial thickness tissues containing only epithelial cells were used in these studies. Tissues for a single experiment were derived from a single donor. Following tissue generation, inserts were cultured in the absence of steroid hormones and grown in phenol red-free media. The apical surface remained at an ALI, while the basal surface was exposed to one of four conditions: media with no added hormones; media with estradiol; media with testosterone; and media with 5-alpha-dihyrotestosterone (DHT). The choice of hormone concentrations used was based on the physiological range of estradiol and testosterone in cis-gender females and cis-gender males, with adjustment for the tissue-to-serum gradient that has been described. Specifically, serum estradiol levels across the ovulatory cycle can range from 30 to 1900 pmol/L [26]. Furthermore, the data suggests that estradiol concentrations in the reproductive tract can be 5 to 8 times higher than serum concentrations, due to local metabolism [27,28]. For testosterone, the level chosen was based on the peak of the normal male physiological range of testosterone that is generally the target for transgender men [29]; unfortunately, there is no data for the female reproductive tract tissue levels of testosterone compared to the serum levels, so we made the assumption that it would have a similar gradient. Finally, because vaginal tissues express both 5α-reductase type 1 and type 2, which convert testosterone locally to DHT, we included a DHT condition, since it has a higher affinity for the androgen receptor than testosterone, and it has been shown to non-transcriptionally activate additional signaling pathways [30,31]. Thus, the hormone levels used for this study are as follows: 17-β estradiol at a concentration of 10 nmol/L (MP Biomedicals, Santa Ana, CA, USA); testosterone enthanate at a concentration of 150 nmol/L (Hikma Pharmaceutical, Cherry Hill Township, NJ, USA); or DHT (Sigma-Aldrich, St. Louis, MO, USA) at a concentration of 15 nmol/L. At the end of 7 days, tissues were fixed overnight in 10% formalin for processing by paraffin embedding and routine hematoxylin and eosin (H&E) staining at MatTek or used in other assays, as described below. Tissue thickness was measured on the 4× magnification images using Image J software (version 1.53). Each hormone condition was always run in triplicates on the same plate. The images shown are from a single representative well from the same experiment. H&E staining was performed in 3 independent experiments (i.e., tissue samples obtained on separate occasions, separated by time).

### 2.2. TEER Measurement and FITC-Dextran Transit

The changes in barrier function were quantified using transepithelial electrical resistance (TEER) measurements, using a Millicell ERS-2 Electrical Resistance System (EMD Millipore Corporation, Burlington, MA, USA). The TEER values (reported in ohm × cm^2^) were calculated by multiplying raw resistance measurements by the area of the tissue (0.6 cm^2^). The barrier function was also tested using FITC-dextran (Sigma), an average molecular weight of 10,000, and translocation across the apical to basal surface. FITC-Dextran was diluted in phenol red-free media at a concentration of 1 mg/mL, and 250 µL was added to the apical surface. After 30 min at 37 °C, 100 µL of the basal media was transferred to a clear bottom black polystyrene microplate and assayed for fluorescence at 490/520 nm excitation/emission. Each hormone condition was always run in triplicates on the same plate. TEER and FITC dextran were performed on 2 independent tissue experiments.

### 2.3. RNA Extraction and RNA-Seq Data Processing and Analysis

Tissues were treated with hormones as described above, and each hormone condition was run in triplicates on the same plate. The total RNA was extracted using the RNAqueous Micro Kit (Invitrogen, Carlsbad, CA, USA). Modifications to the manufacturer’s protocol for the MatTek tissues were as follows. The microporous membrane from the cell culture inserts was cut away using sharp forceps. It was added to lysis buffer and homogenized using a pellet pestle mixer tip. RNA extraction was then performed according to the manufacturer’s instructions.

RNA sequencing, including data analysis, was performed by Arraystar Inc., Rockville, MD, USA. Briefly, 1–2 µg total RNA was used to prepare the sequencing library with the following steps. The total RNA was enriched by oligo (dT) magnetic beads (rRNA removed). RNA-seq library preparation using a KAPA Stranded RNA-Seq Library Prep Kit (Roche Diagnostics, Indianapolis, IN, USA), which incorporates dUTP into the second cDNA strand and renders the RNA-seq library strand-specific, was performed. The completed libraries were qualified with an Agilent 2100 Bioanalyzer (Agilent Technologies, Santa Clara, CA, USA) and quantified by the absolute quantification qPCR method. To sequence the libraries on the Illumina NovaSeq 6000 instrument (Illumina, San Diego, CA, USA), the barcoded libraries were mixed, denatured to single-stranded DNA in NaOH, captured on Illumina flow cell, amplified in situ, and subsequently sequenced for 150 cycles for both ends on an Illumina NovaSeq 6000 instrument.

Image analysis and base calling were performed using Solexa pipeline v1.8 (Off-Line Base Caller software, v1.8). The sequence quality was examined using the FastQC software (version 0.11.7). The trimmed reads were aligned to the reference genome using Hisat2 software (version 2.1.0) [32]. The transcript abundance for each sample was estimated with StringTie [33], and the normalized expression level or FPKM (fragments per kilobase of transcript per million mapped reads) [34] value for known genes and transcripts were calculated with the R package Ballgown (version 2.10.0) [35]. The following cutoffs were used for filtering differentially expressed genes and transcripts: fold change ≥ 1.5, *p*-value ≤ 0.05, and FPKM ≥ 0.5 mean in at least one group. Gene ontology (GO) enrichment analysis of differentially expressed genes was performed using standard GO terms from the Gene Ontology Resource (http://www.geneontology.org, accessed on 26 December 2024). Principal Component Analysis (PCA), Correlation Analysis, Hierarchical Clustering, Gene Ontology (GO), Pathway Analysis, Scatter Plots and Volcano Plots are performed for the differentially expressed genes in R (version 3.5.0) or Python (version 2.7) software for statistical computing and graphics, including standard False Discovery Rate (FDR) correction for multiple testing to calculate a q-value.

### 2.4. Data Deposition

RNA-sequencing data for this publication has been uploaded to Gene Expression Omnibus (GEO) and is available under the dataset name “Effect of cross sex hormones on gene expression in Mattek reconstructed vaginal tissue model” at GSE317796. This dataset includes both raw data in the fastq format and a matrix table of processed data (xlsx format) with the normalized FPKM expression values for known genes from each sample.

## 3. Results

### 3.1. Estradiol Induces Thickening and Proliferation in the Stratum Basale of Vaginal Tissue Compared to Testosterone or DHT Without Altering the Barrier Function

**Figure 1:**
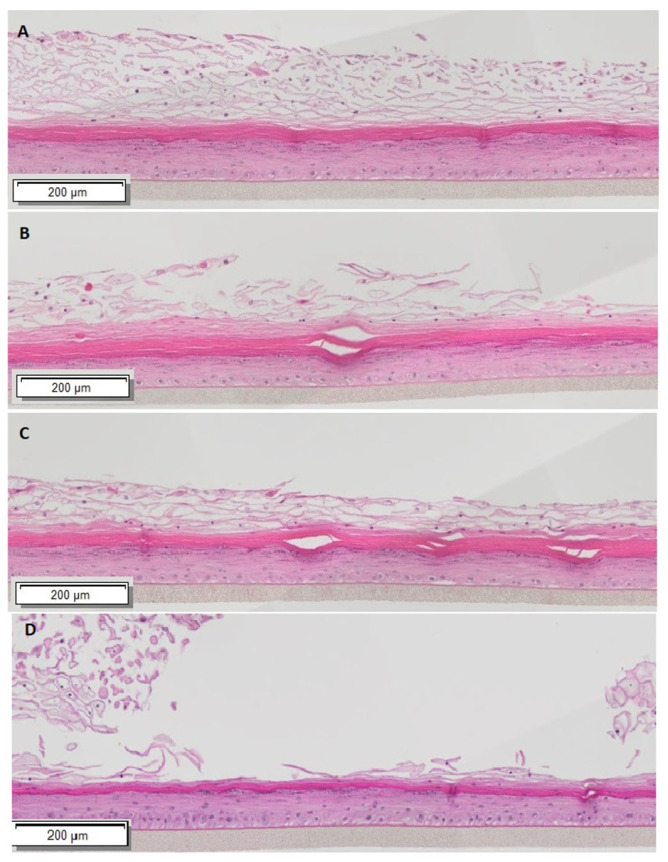
Histology of VEC tissues exposed to sex hormones. MatTek VEC PT tissues were equilibrated in 10 nmol/L estradiol (**A**), 150 nmol/L testosterone (**B**), 15 nmol/L DHT (**C**) or kept without hormone exposure (**D**) for 7 days. At that point, tissues were fixed in formalin and stained by H&E. Shown above are representative images from triplicate samples of a single experiment out of three independent experiments. Original magnification is 4×. Horizontal bar shows 200 µM scale.

In order to confirm the hormone responsiveness of our tissues to the sex hormone concentrations being used, we first carried out histological analysis by H&E staining of the MatTek VEK partial thickness tissues. Estrogen responsiveness was tested using 17β-estradiol, the primary estrogen produced by the ovaries. Androgen responsiveness was tested using testosterone enthanate, an injectable formulation that is often used in testosterone replacement therapy, and 5-alpha-dihyrotestosterone, also known as DHT, which is a metabolite of testosterone synthesized in tissues with a higher binding affinity for the androgen receptor and additional signaling effects [36]. The concentrations used were 4–5-fold higher than the average serum levels, based on the general consensus that tissue levels, while influenced by serum levels, can be higher due to local synthesis and metabolism [27,37,38]. Representative histology images of tissues exposed to estradiol, testosterone, or DHT, as well as tissues with no added hormone, are shown in Figure 1. We found that the estradiol-treated tissues were thicker than the testosterone or DHT-treated tissues overall, in both the lower basal and suprabasal zones where replicating cells would be expected, as well as the stratum corneum. All hormone-treated tissues were also thicker than the no-hormone-treated control tissues. Notably, the estradiol-treated tissues displayed more detachment or sloughing of the stratum corneum compared to the testosterone or DHT tissues. While this observation could just be an artifact of the fixation and staining process, it was seen consistently enough to suggest there could be alterations in the exfoliation of this outer layer under the influence of estradiol, which could impact the barrier function. Tissue thickness, including the thickness of specific layers, was quantified for the three hormone treatment conditions by measuring six sites on each tissue sample using Image J software, and it is summarized in Table 1.

In order to directly compare the barrier function of the epithelial cells under the different hormonal conditions, we next determined the transepithelial electrical resistance (TEER) of the estradiol- or testosterone-treated tissues. When normalized to the pre-hormone-treated tissues, we found that all three hormone conditions resulted in an increase in TEER after 7 days, with the greatest increase seen with DHT treatment (Table 2). Given the histology, this could reflect the sloughing seen in the estradiol-treated tissues or reflect changes in the expression of tight junctions. The increased TEER, however, had no effect on the barrier function as measured by FITC-dextran translocation from the apical to basal side, as none of the tissues, including the no-hormone-treated tissues, had any detectable fluorescence at 490/520 nm in basal media. We concluded that the tissues were hormone responsive, as shown by the histological changes and increased TEER compared to pre-hormone treatment, but we observed no impairment of the barrier function to the passage of FITC-dextran.

### 3.2. Sex Hormone Exposure Modulates Gene Expression in Vaginal Epithelial Cells

**Figure 2:**
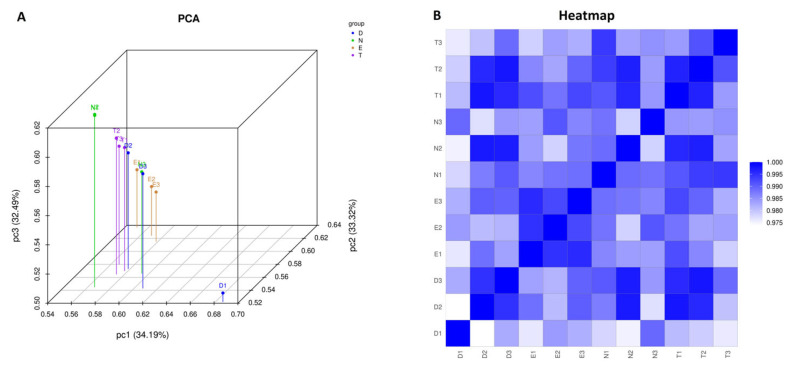
Gene expression level PCA and correlation analysis, grouped by sex hormone exposure. Principal Component Analysis (PCA) was performed with genes that have the ANOVA *p* value ≤ 0.05 on FPKM abundance estimations. Shown in (**A**) is an overview of the clustering of the triplicate samples between the 4 conditions. Shown in (**B**) is the Pearson R2 correlation of gene expression between the 3 biological replicates of each group. The majority of samples fell within the acceptable threshold of >0.92. N: no added hormone. E: estradiol. T: testosterone. D: DHT.

In order to examine the effects of sex hormone exposure on the gene expression profile of the epithelial cells in reconstructed vaginal tissues, we conducted the RNA sequencing of total RNA prepared from tissues grown in no hormones, estradiol, testosterone, or DHT. Analyses of gene expression changes in the tissues exposed to hormones compared to no hormones, revealed that there were a total of 11,120 differentially expressed genes following estradiol exposure; 11,094 with testosterone exposure; and 11,130 with DHT exposure. The results of the Principal Component Analysis (PCA) demonstrated that a distinguishable gene expression profile was identified among the sample groups, based on sex hormone exposure (Figure 2A). The Pearson correlation demonstrated a high correlation between the estradiol samples, with overlap, as expected, between the testosterone and DHT samples (Figure 2B). Importantly, the majority of samples fell within the acceptable threshold, with the exception of one DHT outlier. Because this sample was not found to have technical errors, it was not removed from further data analysis.

#### Estradiol Has a Broad Effect on Gene Expression in Vaginal Tissues Compared to Androgens

**Figure 3:**
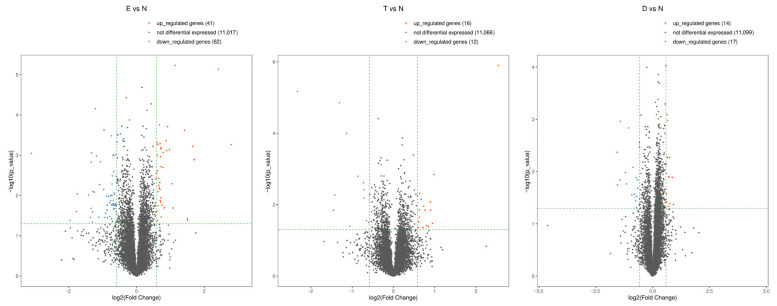
Volcano Plots of differentially expressed genes in response to sex hormone exposure. Pairwise comparisons of RNA-seq were conducted on estradiol- (**left**), testosterone- (**center**), or DHT (**right**)-treated tissues compared to the no-hormone-treated control. The values of the X and Y axes in the Volcano Plots are log2 transformed fold change (*X*-axis) and log10 transformed *p*-values (*Y*-axis) between the two groups. Red/blue dots indicate statistically significant differentially expressed genes with fold change > 1.5 and *p*-value ≤ 0.05. Red: upregulated genes; blue: downregulated genes; gray: non-differentially expressed genes based on cutoff thresholds. N: no hormone. E: estradiol. T: testosterone. D: DHT.

To determine the sex hormone responsiveness of the vaginal tissues, we conducted bulk RNA-seq analysis, comparing hormone treatment to the no-hormone-treated control. Using a log2 Fold Change and *p*-value of ≤0.05 for significance, we found that estradiol treatment upregulated 41 genes and downregulated 62 genes compared to the no-hormone-treated controls. For testosterone, there were 16 upregulated and 12 downregulated genes, and 14 upregulated and 17 downregulated genes for DHT. The Volcano Plots shown in Figure 3 are a visualization method for the quick identification of genes, displaying large magnitude changes which are also statistically significant. Each plot is constructed by mapping −log10 *p*-value on the *y*-axis, and log2 Fold-Change in mRNA between the two experimental groups on the *x*-axis, with significantly upregulated (red) or downregulated (blue) genes shown in color (significance defined as 1.5-fold change, *p*-value ≤ 0.05). This is visually represented by the density of the data points in the upper left and right regions on the Volcano Plots. The top 10 genes that were up- or downregulated in each pairwise comparison are shown in the Supplementary Materials Tables S1–S6, including the associated FDR-adjusted *p*-values.

**Figure 4:**
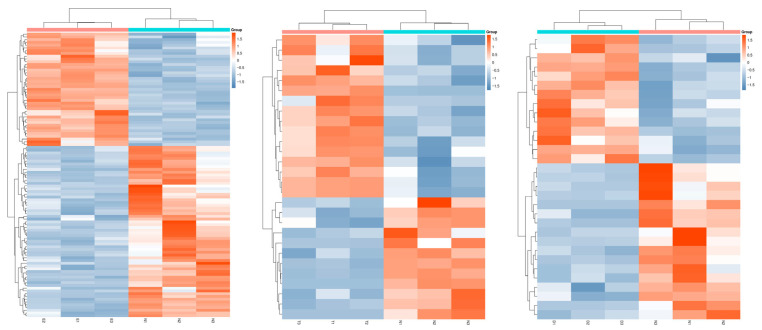
Hierarchical clustering of significantly expressed genes in response to sex hormone exposure. The heatmaps were developed from RNA-seq analysis of significant differentially expressed genes in estradiol- (**left**), testosterone- (**center**), or DHT (**right**)-treated tissues compared to no-hormone-treated controls. In each image, the hormone treatment samples are in columns 1–3, while the no-hormone control samples are in columns 4–6. Each row corresponds to a gene, and each column is a sample. The color represents the relative expression level, with red indicating higher values and blue indicating lower values. N: no hormone. E: estradiol. T: testosterone. D: DHT.

In order to better visualize the pattern of gene expression changes in response to the hormone treatments compared to the no treatment control, we constructed unsupervised hierarchical clustering heatmaps, using the same cutoffs for significance. As shown in Figure 4, there were more genes with altered expression in response to estradiol treatment compared to the two androgens, testosterone or DHT, although all three treatments resulted in a unique profile compared to the no-hormone-treated control tissues.

Together, these results demonstrate broad changes in gene expression in the vaginal epithelium under the influence of estradiol exposure. In contrast, the testosterone and DHT tissues, while also altering the steady state gene expression profile of the tissues, have greater similarity to the no-hormone-treated control tissues.

### 3.3. Estrogen- and Androgen-Dominant Vaginal Tissues Display Unique Gene Expression Signatures

**Figure 5:**
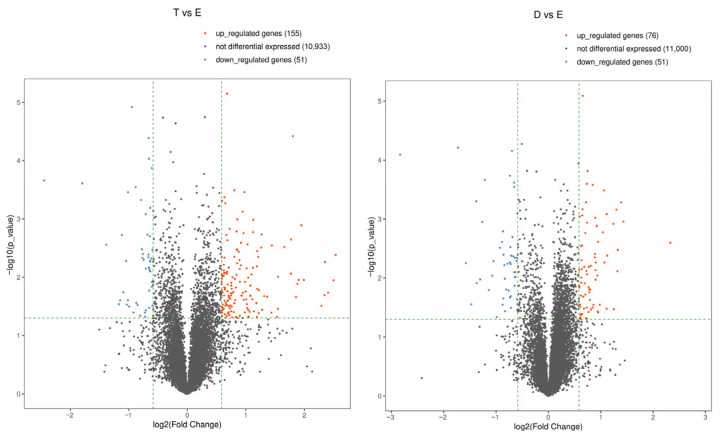
Volcano Plots of differentially expressed genes in estrogen vs. androgen exposed vaginal tissues. Pairwise comparisons of RNA-seq were conducted on testosterone vs. estradiol (**left**) and DHT- vs. estradiol (**right**)-treated tissues. The values of the X and Y axes in the Volcano Plots are log2 transformed fold change (*X*-axis) and log10 transformed *p*-values (*Y*-axis) between the two groups. Red/Blue dots indicate statistically significant differentially expressed genes with fold change > 1.5 and *p*-value ≤ 0.05. Red: upregulated genes; Blue: downregulated genes; Gray: non-differentially expressed genes based on cutoff thresholds. E, estradiol. T, testosterone. D, DHT.

While comparisons between specific sex hormone treatments and the untreated controls are useful, the no-hormone control tissue is not physiologically relevant, as tissues are exposed to a variety of hormones, including combinations of hormones, in vivo. Thus, we chose to more directly examine the effects of estrogens, using the estradiol-treated tissues, on androgens, using the testosterone or DHT-treated tissues, to better understand how a single dominant sex hormone might alter gene expression in the vaginal epithelium. The Volcano Plots shown in Figure 5 identify the genes displaying large magnitude changes (>1.5-fold), which are also statistically significant (*p*-value ≤ 0.05). When compared to estradiol, testosterone led to the upregulation of 155 and downregulation of 51 genes, with 76 upregulated and 51 downregulated genes in response to DHT. Thus, the estrogen vs. androgen treatments had more significant changes compared to each other than compared to the no-hormone treatment control (Figure 3). Notably, the changes in response to testosterone vs. estradiol were distinct from DHT vs. estradiol, despite both hormones binding to the androgen receptor. The top 10 genes that were up- or downregulated in each pairwise comparison are shown in the Supplementary Materials Tables S7–S10.

#### mRNA Function Enrichment Analysis in Estrogen- vs. Androgen-Dominant Vaginal Tissues

**Figure 6:**
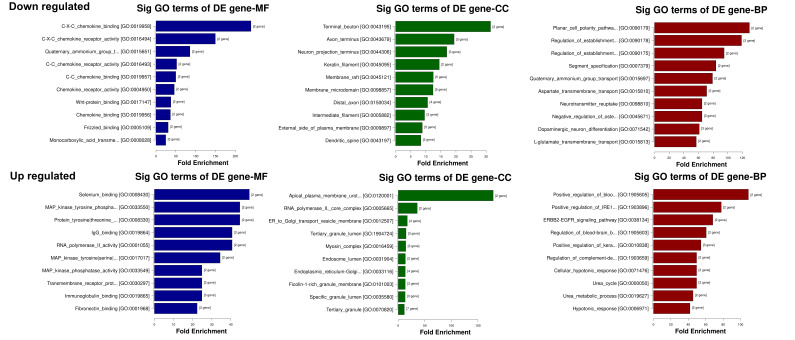
Top 10 enriched GO terms (BP CC MF) for testosterone- vs. estradiol-treated tissues. The top 10 GO terms for differentially expressed genes, categorized by molecular function (MF), cellular component (CC) and biological processes (BP). **Top row**: downregulated pathways. **Bottom row**: upregulated pathways. Terms are ordered from top to bottom by increasing *p*-value (−log10 scaled).

**Figure 7:**
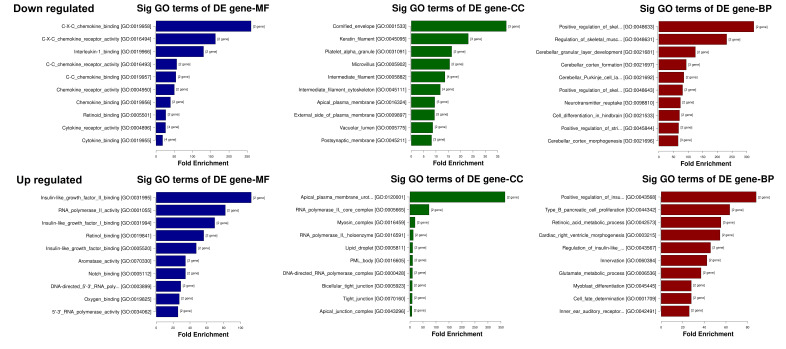
Top 10 enriched GO terms (BP CC MF) for DHT- vs. estradiol-treated tissues. The top 10 GO terms for differentially expressed genes, categorized by molecular function (MF), cellular component (CC) and biological processes (BP). **Top row**: downregulated pathways. **Bottom row**: upregulated pathways. Terms are ordered from top to bottom by increasing *p*-value (−log10 scaled).

In order to identify the biological relevance of groups of differentially expressed genes in response to either estrogen or androgen dominance, we utilized Gene Ontology (GO) analysis to determine if specific GO terms are more likely to be associated with the differentially expressed genes that we identified by the pairwise comparisons of estradiol vs. testosterone and estradiol vs. DHT. The GO analysis comprises three structured relationships of defined terms that describe gene product attributes: Biological Process (BP), Molecular Function (MF), and Cellular Component (CC). Table 2 and Table 3 show the Top 5 significantly enriched GO results for the 3 categories testosterone vs. estradiol (Table 3) and DHT vs. estradiol (Table 4). The Top 10 enriched GO Terms for the hormone treatments are depicted in Figure 6 and Figure 7. Notable findings include relative changes in expression of pathways involved in chemokine receptor activity, with lower expression found in testosterone- and DHT-treated tissues compared to estradiol-treated tissues, as well as relative downregulation of cell differentiation and development pathways.

## 4. Discussion

The human vaginal epithelium is a multilayered stratified squamous epithelium that provides a physical and immunological barrier to sexually transmitted infections [10,39]. It must both tolerate endogenous flora while at the same time acting as a sentinel to invading pathogens. The female reproductive tract is also a dynamic tissue, responding to the endogenous and exogenous reproductive hormonal milieu [40]. Despite the importance of hormonal changes on the tissue function, many questions remain unanswered. Human studies are difficult to undertake, mouse models must account for endogenous hormones in the estrus cycle, and in vitro studies often utilize transformed or immortalized cells grown in 2D cultures. Our study is unique in that we have taken advantage of reconstructed vaginal epithelial cell tissues developed from primary human cells.

The hormonal responsiveness of the reconstructed tissues to estradiol has been published by others [13], but ours is the first to additionally examine the effects of androgens on the vaginal epithelial tissue, specifically testosterone and DHT. We expected to see tissue thinning under the influence of the androgens compared to estradiol on histological analysis. While the differences in tissue thickness were statistically significant between the sex hormone conditions, testosterone and DHT treatment did not result in obvious detrimental effects on the barrier function of the tissues compared to estradiol treatment. In fact, the DHT, and to a lesser extent, testosterone-treated tissues displayed higher TEER compared to the estradiol-treated tissues. However, there were some consistent phenotypic changes observed in the tissues of unclear significance. The stratum corneum of the vagina normally consists of flattened cells lacking nuclei and organelles, loosely connected without tight intercellular junctions [10]. We found qualitative changes in the H&E staining in the estradiol-treated tissues that suggested increased tissue exfoliation above a more tightly packed layer of cornification, although it remains unclear if this would have any impact on tissue barrier function or resilience over time, since none of the treatment regimens resulted in decreased TEER or allowed for the passage of FITC-dextran dye. There are limited studies suggesting that the use of topical testosterone can relieve some vaginal atrophy symptoms in women with breast cancer who are unable to take exogenous estrogens, and it has also been studied as a treatment for sexual dysfunction in post-menopausal women and women on aromatase inhibitors [41,42,43]. Thus, it was reassuring that we observed no obvious impairment in barrier function as a result of androgen exposure. However, further studies on the long-term effects of testosterone and other androgens on the vaginal mucosa, and specifically the stratum corneum where immunoglobulins and antimicrobial peptides are retained [10,44], seem warranted to further assess its safety and efficacy.

To that end, our RNA sequencing data could be a starting point for further studies. The hierarchical clustering on the sex-hormone-treated tissues demonstrates distinct gene expression profiles in response to estradiol and the androgens, testosterone and DHT. While the individual gene expression analyses yielded q-values > 0.05, which is typically considered the cutoff for significance, for these exploratory studies with only three biological replicates per condition, it is reasonable to expand the cutoff, although it did limit our power to detect smaller changes. Moreover, pathway analysis using gene set enrichment analysis is still valid to identify subtle coordinated changes across multiple genes in a specific pathway. Using this approach, we identified a number of pathways that were differentially regulated, and two specifically stood out to us as being relevant in the lower female reproductive tract. First, some of the enriched GO terms included the upregulation of genes involved in tight junctions or apical junctions in response to DHT compared to estradiol, and keratinocyte proliferation in response to testosterone compared to estradiol. These differences could align with the phenotypic changes observed in the H&E tissue staining described above, as well as the increased TEER.

A second notable finding is the downregulation of multiple gene sets involved in chemokine signaling in response to both testosterone and DHT compared to estradiol. Virtually every mammalian cell type is capable of secreting chemokines, which are widely expressed, while their receptors are more specific in expression, with many localized to specific leukocytes, such as neutrophils, monocytes, mast cells, eosinophils, and T and B cells (reviewed in [45]). Chemokine expression has been associated with numerous pathogens, where they are thought to aid in host resistance by recruiting specific leukocyte subpopulations to the site of infection. This is particularly true for many bacterial infections, where resistance to infection depends on recruitment of neutrophils and monocytes. A number of viruses have also been shown to actively evade chemokines. Thus, any change in the expression of chemokines in the lower female reproductive tract could have the potential to impair host defenses when exposed to sexually transmitted bacterial and viral pathogens. For example, our analysis identified decreased expression of genes involved in both C-X-C and C-C chemokine binding and/or receptor activity. As we only examined the steady-state gene expression under specific hormone conditions, we do not know if this would alter tissue responses to pathogen-associated inflammatory ligands. If it were to blunt chemokine receptor expression or signaling in cells, it could impair the ability of the mucosal surface to promptly respond to infections, reducing the necessary recruitment of immune cells to the site of an infection that could impact both the innate and adaptive immune response. Thus, blunted chemokine responses, combined with potential alterations in sequestering of antimicrobial peptides in the outer stratum corneum, could impair immune defenses in a testosterone-dominant vaginal microenvironment.

Our model does, however, have some limitations that impact the applicability of our findings to intact vaginal tissue. First, our model only utilized vaginal epithelial cells, so we do not have a full understanding of the interplay between immune cells and fibroblasts in the submucosa with the epithelial cells under these various hormone conditions. Such studies could be undertaken with the repopulation of the tissues with a layer of fibroblasts, but it would be more difficult to recapitulate the presence of immune cells such as tissue macrophages, dendritic cells, and lymphocytes, given our limited understanding of the density and activation status of these cells and an inability to really examine the recruitment of immune cells. In either case, single-cell sequencing would be needed to examine specific cell population responses. Our use of single hormone conditions at the higher end of the normal range also vastly oversimplifies the complicated ovulatory cycle of reproductive aged women where estrogen levels vary widely, with or without progesterone, which we did not include. This is further complicated by the presence of low circulating testosterone levels, even in healthy females. However, by utilizing data we have on the effects of isolated hormones, one might be able to develop computer modeling to then examine the interplay of various hormones, possibly with the addition of other cell types to the mix that could predict a multicellular response under specific conditions. Finally, an unanswered question that this model was not designed to address is what the effects of sex hormone exposure on the vaginal microbiome are. For example, changes in mucins and glycogen production along the apical surface of the vaginal mucosa under the response of varying hormone conditions likely will have an impact on the maintenance of a healthy vaginal microbial community. Unfortunately, such fastidious organisms can be difficult to maintain in culture without damaging the cell culture.

Ultimately, further data on the effects of both estrogens and androgens on the female reproductive tract will inform the development of strategies to protect women experiencing menopause and individuals undergoing gender-affirming hormone therapy to lessen the risk of long-term health risks, including the loss of a healthy vaginal microbiome, the acquisition of HIV and other STIs during vaginal intercourse, and symptoms of vaginal atrophy. Ours is just one of hopefully many studies focused on women’s health research to come.

## Figures and Tables

**Table 1:** Thickness of VEC tissues exposed to estradiol, testosterone, or DHT.

	Estradiol		Testosterone			DHT		
	Mean, µM	SD	Mean, µM	SD	*p*-Value vs. E	Mean, µM	SD	*p*-Value vs. E
Full tissue	256.62	17.43	202.53	5.90	<0.0001	181.47	17.80	<0.0001
Basal and suprabasal	86.96	6.80	54.99	3.73	<0.0001	65.81	5.09	<0.0001

*p*-value based on two-tailed, unpaired *t*-test.

**Table 2:** Fold increase TEER with hormone treatment.

Fold Change Ω × cm^2^	Estradiol	Testosterone	DHT
mean	2.27	3.76	5.38
SD	0.55	0.88	0.92

*p* < 0.5, one-way ANOVA.

**Table 3:** Top 5 significantly enriched GO results for testosterone vs. estradiol.

T vs. E DOWN MF					
ID	Term	Count	*p*-value	fdr	Fold-Enrichment
GO:0005515	protein binding	42	8.86035 × 10^−8^	2.73785 × 10^−5^	1.748136797
GO:0004896	cytokine receptor activity	4	2.41112 × 10^−5^	0.002816584	23.90285714
GO:0019958	C-X-C chemokine binding	2	2.73455 × 10^−5^	0.002816584	239.0285714
GO:0016494	C-X-C chemokine receptor activity	2	7.63219 × 10^−5^	0.005895865	149.3928571
GO:0140375	immune receptor activity	4	0.000132945	0.008215994	15.42119816
T vs. E DOWN CC					
ID	Term	Count	*p*-value	fdr	Fold-Enrichment
GO:0005576	extracellular region	21	2.13527 × 10^−7^	2.30893 × 10^−5^	3.328357424
GO:0071944	cell periphery	25	2.45631 × 10^−7^	2.30893 × 10^−5^	2.787243876
GO:0016020	membrane	29	3.96827 × 10^−6^	0.000176954	2.14544265
GO:0009986	cell surface	9	4.01943 × 10^−6^	0.000176954	7.118163934
GO:0005886	plasma membrane	22	4.85319 × 10^−6^	0.000176954	2.653051158
T vs. E DOWN BP					
ID	Term	Count	*p*-value	fdr	Fold-Enrichment
GO:0048856	anatomical structure development	26	7.48674 × 10^−10^	1.13195 × 10^−6^	3.500896044
GO:0030154	cell differentiation	22	1.37875 × 10^−9^	1.13195 × 10^−6^	4.157443695
GO:0050896	response to stimulus	31	7.2812 × 10^−9^	3.38501 × 10^−6^	2.615461126
GO:0032502	developmental process	26	8.24606 × 10^−9^	3.38501 × 10^−6^	3.137505884
GO:0048731	system development	20	2.42382 × 10^−8^	7.70196 × 10^−6^	3.970367165
T vs. E UP MF					
ID	Term	Count	*p*-value	fdr	Fold-Enrichment
GO:0005515	protein binding	104	7.23443 × 10^−13^	3.06016 × 10^−10^	1.619139426
GO:0030545	signaling receptor regulator activity	12	9.21425 × 10^−6^	0.001948813	4.755725191
GO:0048018	receptor ligand activity	11	1.81141 × 10^−5^	0.002173378	4.859110521
GO:0030546	signaling receptor activator activity	11	2.0552 × 10^−5^	0.002173378	4.792806869
GO:0003824	catalytic activity	46	4.375 × 10^−5^	0.003701252	1.74653947
T vs. E UP CC					
ID	Term	Count	*p*-value	fdr	Fold-Enrichment
GO:0005576	extracellular region	62	1.73085 × 10^−21^	4.50021 × 10^−19^	3.639473727
GO:0005615	extracellular space	55	5.37315 × 10^−21^	6.9851 × 10^−19^	4.082423049
GO:0031982	vesicle	49	5.34121 × 10^−14^	4.62905 × 10^−12^	3.154047035
GO:0070062	extracellular exosome	35	1.13319 × 10^−13^	7.36572 × 10^−12^	4.303227999
GO:0016020	membrane	77	2.01074 × 10^−13^	1.04559 × 10^−11^	2.109822274
T vs. E UP BP					
ID	Term	Count	*p*-value	fdr	Fold-Enrichment
GO:0032502	developmental process	58	6.31214 × 10^−14^	1.31372 × 10^−10^	2.679324436
GO:0050896	response to stimulus	70	1.23703 × 10^−13^	1.31372 × 10^−10^	2.260844673
GO:0048856	anatomical structure development	51	1.07556 × 10^−11^	7.61498 × 10^−9^	2.628827888
GO:0044419	biological process involved in interspecies interaction	24	4.35158 × 10^−9^	2.31069 × 10^−6^	4.062655376
GO:0048583	regulation of response to stimulus	40	9.90269 × 10^−9^	3.68765 × 10^−6^	2.563455882

ID: Gene Ontology ID; Term: The name of Gene Ontology terms; Count: The number of genes associated with the ID; *p*-value: The Fisher’s exact test *p*-value; fdr: The FDR-adjusted *p*-value.

**Table 4:** Top 5 significantly enriched GO results for DHT vs. estradiol.

D vs. E DOWN MF					
ID	Term	Count	*p*-value	fdr	Fold-Enrichment
GO:0005515	protein binding	37	4.2429 × 10^−6^	0.00108194	1.676916409
GO:0004896	cytokine receptor activity	4	1.71339 × 10^−5^	0.001957271	26.02755556
GO:0019958	C-X-C chemokine binding	2	2.30267 × 10^−5^	0.001957271	260.2755556
GO:0016494	C-X-C chemokine receptor activity	2	6.42857 × 10^−5^	0.003743614	162.6722222
GO:0019955	cytokine binding	4	7.34042 × 10^−5^	0.003743614	17.95003831
D vs. E DOWN CC					
ID	Term	Count	*p*-value	fdr	Fold-Enrichment
GO:0005576	extracellular region	23	1.50142 × 10^−9^	2.37224 × 10^−7^	3.878025368
GO:0005737	cytoplasm	36	1.13992 × 10^−6^	6.33667 × 10^−5^	1.84057672
GO:0071944	cell periphery	23	1.20317 × 10^−6^	6.33667 × 10^−5^	2.727940815
GO:0016020	membrane	28	2.79648 × 10^−6^	0.000110461	2.203682839
GO:0005615	extracellular space	16	6.842 × 10^−6^	0.000216207	3.411231639
D vs. E DOWN BP					
ID	Term	Count	*p*-value	fdr	Fold-Enrichment
GO:0048856	anatomical structure development	27	1.5283 × 10^−12^	1.83549 × 10^−9^	4.142831365
GO:0032502	developmental process	27	2.15632 × 10^−11^	1.29487 × 10^−8^	3.712808842
GO:0008544	epidermis development	9	2.87168 × 10^−10^	9.8263 × 10^−8^	21.58848218
GO:0048513	animal organ development	19	3.2727 × 10^−10^	9.8263 × 10^−8^	5.308263748
GO:0030154	cell differentiation	21	4.68119 × 10^−10^	1.12442 × 10^−7^	4.522208839
D vs. E UP MF					
ID	Term	Count	*p*-value	fdr	Fold-Enrichment
GO:0005515	protein binding	53	5.97511 × 10^−8^	1.5595 × 10^−5^	1.662971159
GO:0046914	transition metal ion binding	11	3.65005 × 10^−5^	0.004763319	4.37356236
GO:0031995	insulin-like growth factor II binding	2	0.000134696	0.011718566	112.6192308
GO:0001055	RNA polymerase II activity	2	0.000263446	0.01574809	81.9048951
GO:0046872	metal ion binding	21	0.000301688	0.01574809	2.179224922
D vs. E UP CC					
ID	Term	Count	*p*-value	fdr	Fold-Enrichment
GO:0016020	membrane	36	2.11382 × 10^−6^	0.000422764	2.017657664
GO:0120001	apical plasma membrane urothelial plaque	2	9.8185 × 10^−6^	0.000581487	365.4949495
GO:0071944	cell periphery	27	1.08949 × 10^−5^	0.000581487	2.280472262
GO:0005737	cytoplasm	45	1.16297 × 10^−5^	0.000581487	1.638392156
GO:0005576	extracellular region	21	3.75062 × 10^−5^	0.001500248	2.521482897
D vs. E UP BP					
ID	Term	Count	*p*-value	fdr	Fold-Enrichment
GO:0032502	developmental process	29	2.95747 × 10^−8^	3.5046 × 10^−5^	2.81109449
GO:0050896	response to stimulus	32	1.80302 × 10^−6^	0.001068288	2.168716576
GO:0048856	anatomical structure development	24	3.86491 × 10^−6^	0.001526641	2.595872476
GO:0006629	lipid metabolic process	11	8.87503 × 10^−6^	0.002629228	5.078166795
GO:0006082	organic acid metabolic process	9	1.36098 × 10^−5^	0.002710115	6.189797005

ID: Gene Ontology ID; Term: The name of Gene Ontology terms; Count: The number of genes associated with the ID; *p*-value: The Fisher’s exact test *p*-value; fdr: The FDR-adjusted *p*-value.

## Data Availability

The data presented in this study are openly available in [GEO] at [https://www.ncbi.nlm.nih.gov/geo/], reference number [GSE317796].

## Supplementary Materials

The following supporting information can be downloaded at: https://www.mdpi.com/article/10.3390/cells15090745/s1, Table S1: Top 10 genes upregulated E vs. N tissues; Table S2: Top 10 genes down regulated E vs. N tissues; Table S3: Top 10 genes upregulated T vs. N tissues; Table S4: Top 10 genes down regulated T vs. N tissues; Table S5: Top 10 genes upregulated D vs. N tissues; Table S6: Top 10 genes down regulated D vs. N tissues; Table S7: Top 10 genes upregulated T vs. E tissues; Table S8: Top 10 genes down regulated T vs. E tissues; Table S9: Top 10 genes upregulated DHT vs. E tissues; Table S10: Top 10 genes down regulated DHT vs. E tissues.

## Abbreviations

The following abbreviations are used in this manuscript:

BP	Biological Process
CC	Cellular Component
DHT	5-alpha-dihyrotestosterone
FDR	False Discovery Rate
FPKM	Fragments Per Kilobase of Transcript Per Million Mapped Reads
GO	Gene Ontology
H&E	Hematoxylin and Eosin
MF	Molecular Function
PCA	Principal Component Analysis
SD	Standard Deviation
