# Supplementary material for: Epithelial Changes in the Testosterone-Dominant Vagina: Implications for Menopause, Transgender Care, and Beyond
Source: Cells. 2026 Apr 22;15(9):745. doi: 10.3390/cells15090745 (PMC13163056; doi:10.3390/cells15090745)
Supplement: Supplementary file 1 [file cells-15-00745-s001.zip › cells-4161415-supplementary.pdf]

Supplemental Data

Table S1: Top 10 genes upregulated E vs N tissues

Gene Name	Fold Change	p-value*	q-value**
NTS	6.928248098	0.000540889	0.214856223
AC023055.1	5.311007451	7.3094E-06	0.046919047
ANPEP	3.258052706	0.001282313	0.237121151
STC1	3.149095984	0.000602494	0.214856223
MFSD14A	2.839899223	0.041065804	0.51564425
KRT25	2.834614951	0.037630387	0.506379828
SPINK9	2.673028907	0.000238875	0.180392869
KRT9	2.197661788	5.9219E-06	0.046919047
DDX19A	2.106037564	0.02044487	0.456717804
GREB1	2.076351999	0.005096358	0.330238897

*, p-value of the F-statistic for gene
**, q-value or FDR adjusted p-value

Table S2: Top 10 genes down regulated E vs N tissues

Gene Name	Fold Change	p-value*	q-value**
KRT4	0.116883335	0.000891783	0.228974208
UGT1A4	0.256718299	0.04142596	0.51564425
S100A4	0.292775967	0.024799053	0.465221027
CRNN	0.297732592	0.009008239	0.375012671
SERPINB12	0.375671095	0.009738947	0.384009989
TMEM265	0.388620469	0.034692406	0.4951426
UPK3BL1	0.394765328	0.020719696	0.456717804
UHMK1	0.39598937	0.001482114	0.240852854
ANXA10	0.397936225	0.000872283	0.228538057
KRT15	0.40483408	0.045587596	0.526871192

*, p-value of the F-statistic for gene
**, q-value or FDR adjusted p-value

Table S3: Top 10 genes upregulated T vs N tissues

Gene Name	Fold Change	p-value*	q-value**
AC023055.1	5.843251485	1.27221E-06	0.016326282
NABP2	1.978022504	0.001429887	0.662784681
FLRT3	1.924369631	0.03331295	0.945854663
AREGB	1.866680057	0.014153018	0.852510332
PPP2R5D	1.862841245	0.00842579	0.768485352
ITGA2	1.798892333	0.041072169	0.945854663
FOXQ1	1.745610197	0.038818967	0.945854663
AREG	1.699762147	0.014166197	0.852510332
ERCC5	1.662722892	0.011074681	0.824020978
FABP3	1.645138825	0.042873621	0.945854663

*, p-value of the F-statistic for gene
**, q-value or FDR adjusted p-value

Table S4: Top 10 genes down regulated T vs N tissues

Gene Name	Fold Change	p-value*	q-value**
BIVM-ERCC5	0.199373636	6.75734E-06	0.028905669
YTHDF2	0.364081886	0.01414366	0.852510332
PEA15	0.374170486	0.005328099	0.760170844
CTDSPL	0.404158786	1.40217E-05	0.04498505
TSKU	0.453849293	0.000100052	0.213994679
ID3	0.480367629	0.039974578	0.945854663
IRAK1	0.55043536	0.001587666	0.662784681
FLJ20373	0.604189679	0.00243433	0.704173906
DLX3	0.607432889	0.006487992	0.760170844
AMFR	0.609515786	0.003814678	0.760170844

*, p-value of the F-statistic for gene
**, q-value or FDR adjusted p-value

Table S5: Top 10 genes upregulated D vs N tissues

Gene Name	Fold Change	p-value*	q-value**
CHURC1-FNTB	1.8824379	0.042529633	0.657657797
PWP2	1.819600132	0.012904446	0.656235964
MT1E	1.701911773	0.041062412	0.657657797
OXLD1	1.669021505	0.005359157	0.656235964
EIF2A	1.631502126	0.012744779	0.656235964
CENPW	1.630958177	0.012588961	0.656235964
COMMD6	1.629023926	0.012794937	0.656235964
ASNSD1	1.60138781	0.04600015	0.657657797
PELP1	1.575856762	0.001048074	0.589748304
MPDU1	1.558893362	0.000817517	0.589748304

*, p-value of the F-statistic for gene
**, q-value or FDR adjusted p-value

Table S6: Top 10 genes down regulated D vs N tissues

Gene Name	Fold Change	p-value*	q-value**
PEA15	0.337027431	0.004265481	0.656235964
TMEM265	0.341053686	0.01793826	0.656235964
TEN1-CDK3	0.368625864	0.014508534	0.656235964
YTHDF2	0.371289402	0.001090906	0.589748304
HNRNPUL2-BSCL2	0.434778875	0.010380108	0.656235964
TSKU	0.44947566	0.017223456	0.656235964
AC069257.3	0.479030135	0.001462644	0.589748304
IGF2	0.528549246	0.027843993	0.656235964
AQP5	0.547613907	0.008231616	0.656235964
RNF225	0.574934451	0.026853948	0.656235964

*, p-value of the F-statistic for gene
**, q-value or FDR adjusted p-value

Table S7: Top 10 genes upregulated T vs E tissues

Gene Name	Fold Change	p-value*	q-value**
PIP	5.799584382	0.004144957	0.303953127
WFDC2	5.658949952	0.011264541	0.368975777
GDF15	5.304604571	0.018303703	0.411331667
KRT4	5.107458162	0.005494494	0.311618238
MUC21	5.07247621	0.020216316	0.415837054
CEACAM7	4.901703321	0.030829941	0.440464475
CLCA4	3.976973907	0.011123509	0.368975777
MSMB	3.863981026	0.001282856	0.246940173
S100A4	3.745252712	0.011128453	0.368975777
TMPRSS11B	3.688856175	0.012965596	0.385129983

*, p-value of the F-statistic for gene
**, q-value or FDR adjusted p-value

Table S8: Top 10 genes down regulated T vs E tissues

Gene Name	Fold Change	p-value*	q-value**
NTS	0.183174292	0.000219813	0.178955986
ANPEP	0.287793542	0.000245767	0.178955986
MFAP3	0.383313546	0.00275363	0.271333912
STC1	0.444512663	0.028906446	0.436648014
ACKR3	0.451043423	0.024921369	0.420694898
SPINK9	0.460757664	0.001889152	0.250622385
DDX19A	0.468839128	0.018117774	0.411331667
GREB1	0.484382649	0.005255225	0.311618238
TRIM47	0.488289554	0.029341748	0.436975199
AQP5	0.494956677	0.024764633	0.420694898

*, p-value of the F-statistic for gene
**, q-value or FDR adjusted p-value

Table S9: Top 10 genes upregulated DHT vs E tissues

Gene Name	Fold Change	p-value	q-value
KRT4	5.025837388	0.002520792	0.317147623
ADH7	2.698592713	0.001103162	0.228631386
LCE5A	2.620753071	0.000522961	0.215518301
SERPINB12	2.502496861	0.003337425	0.33522872
UPK3BL1	2.492184501	0.007661113	0.361809229
UPK1B	2.459876875	0.000692753	0.215518301
TEN1	2.378417305	0.033677188	0.47953335
UHMK1	2.361788052	0.001212036	0.229163927
ANXA10	2.179755372	0.004173518	0.347150822
RNF4	2.164899792	0.000825375	0.215518301

Table S10: Top 10 genes down regulated DHT vs E tissues

Gene Name	Fold Change	p-value	q-value
NTS	0.140321016	8.08261E-05	0.19947646
ANPEP	0.30235676	6.16358E-05	0.19947646
AQP5	0.335250655	0.005584138	0.352586425
CYP26B1	0.360087506	0.027887083	0.469706234
SPINK9	0.384937691	0.000500247	0.215518301
TEN1-CDK3	0.386667089	0.012211757	0.406752733
TCHH	0.405098132	0.010530594	0.386288338
FP15737	0.416467302	0.001120306	0.228631386
GSKIP	0.431037904	0.000215992	0.19947646
ACKR3	0.453809017	0.016030074	0.426662055
